# R&D Implementation in a Department of Laboratory Medicine and Pathology: *A Systematic Review Based on Pharmaceutical Companies*

**DOI:** 10.5539/gjhs.v7n4p70

**Published:** 2014-12-31

**Authors:** Joseph Feulefack, Consolato Sergi

**Affiliations:** 1 Department of Lab. Medicine and Pathology, University of Alberta, 8440 – 112 St., Edmonton, Alberta T6G 2B7, Canada; 2 Stollery Children’s Hospital, University of Alberta, 8440 – 112 St., Edmonton T6G 2B7, Alberta, Canada

**Keywords:** pharmaceutical companies, R&D Implementation, R&D investments, systematic review

## Abstract

A systematic literature review on pharmaceutical companies may be a tool for guiding some procedures of R&D implementation in a department of Laboratory Medicine and Pathology. The use of pharmaceutical companies for this specific analysis arises from less variability of standards than healthcare facilities. In this qualitative and quantitative analysis, we focused on three useful areas of implementation, including R&D productivity, commercialization strategies, and expenditures determinants of pharmaceutical companies. Studies and reports of online databases from 1965 to 2014 were reviewed according to specific search terms. Initially, 218 articles and reports were found and examined, but only 91 were considered appropriate and used for further analysis. We identified some suggested implementation strategies relevant for marketing to enhance companies’ own R&D strategies; such as reliability of companies on “sourcing-in” R&D facilities and “think-tank” events. Regardless of the study and of the country, cash flow and profitability always positively influenced R&D expenditure, while sales and firm size did not. We consider that handling R&D determinants should require caution. It seems critical that implementation of R&D systems is directly related with productivity, if it reflects dual embodiment of efficiency and effectiveness. Scrutinizing the determinants of R&D expenditures emphasizes significant factors that are worth to highlight when planning an R&D investment strategy. Although there is no receipt fitting every situation, we think that health care plan makers may find relevant data in this systematic review in creating an initial implementation framework.

## 1. Introduction

One of the major challenges of the pharmaceutical industry today is facing profits’ decline due to a slowdown in drug production, increase of costs, and escalating competition. A number of studies have emphasized the positive impact of firms’ R&D investment on output (Danzon, 2006; Kothari et al., 2002). It has been further suggested that R&D investment might generate remarkable returns (International Federation of Pharmaceutical Manufacturers & Associations (IFPMA), 2012). However, a number of factors may cut such investments. Indeed, without disregarding the importance of a strong asset, which is crucial for the economic prosperity of a firm, investment for developing new products and processes is likely to heighten sales, productivity, and commercial value.

The decline in the profitability of pharmaceutical companies has put pressure on their R&D strategies and may even jeopardise further improvements in patient care. A decrease in profits may narrow down innovation incentives, thereby compromising society’s access to the novelty of treatments. Setbacks in firms’ eagerness to develop new drugs will fast translate into lack of medicines that can generate additional savings by reducing costs in other areas of healthcare (i.e. the length of stay in hospital). The release of caregivers who can resume work is another source of additional savings owing to new drug development (Thirumurthy et al., 2008).

In consideration of the above facts, paying due attention to the dropping level of R&D investment by pharmaceutical companies becomes worthwhile. Especially, in the Canadian setting where most of the patented pharmaceutical firms’ source of research funds remains essentially intramural (Industry Canada, 2013), precautions should be put in place to avoid unnecessary R&D budget cuts. In fact, Canadian pharmaceutical companies experienced a decrease in their R&D activities budget in the past. Their R&D spending dropped on average by 15.6% from 2001 to 2012 (Industry Canada, 2013), despite the rising costs of generating new drugs amid the global pharmaceutical market (Dimasi et al., 2003; Dimasi & Grabowski, 2007; International Federation of Pharmaceutical Manufacturers & Associations (IFPMA), 2012). Overall R&D business expenditures by Canadian pharmaceutical companies (CPC) drifted below $1 billion in 2011(Industry Canada, 2013), while pharmaceutical manufacturers in USA rose their R&D budget since the beginning of the century (IFPMA, 2012)

It may be possible that, unless alternative measures are taken, the decrease in investments will further drag down profits (Archarungroj & Yasuo, 1999; Scherer, 2001). Thus, it seems that strategies have to be put in place to avoid unacceptable R&D cuts that result in reducing drug productivity potentially worsened by poor marketing and sales. Our hypothesis is that R&D investments may indeed be necessary and help enhancing the power of the company. Implementation of an R&D strategy that builds on an effective cost handling and refined commercialization for improved profits may open avenues for mitigating escalating health care costs.

Therefore, a systematic review to find which R&D strategies work best is timely. Actually, systematic literature reviews are enlightening guides for successful implementation initiatives (Grol & Jones, 2000). Such reviews help to advance policy-making procedure based on approved, peer-reviewed and/or sounded information (Hagen-Zanker et al., 2012). We focused on pharmaceutical companies’ R&D, because of more homogeneity and structure of these organizations. In addition, the quality standards are probably mostly applicable across the world. A substantial amount of research undertaken by pharmaceutical companies is often extramural involving university and hospital laboratories (Industry Canada, 2013).

Our objective was to conduct a systematic literature review as a tool for guiding the procedures of R&D implementation in diagnostic laboratory facilities. Specific attention in this review is paid to three areas of use to a successful implementation, including productivity, commercialization strategies, and cost-effectiveness of R&D. The next section is the methodology (section 2), followed by the results (section 3) pointing out relevant literature sources and options relative to the three areas of use to a successful implementation. Further, the results present the proxies and explanatory factors of R&D expenditure. Finally, section 4 discusses an implementation framework in light of the reviewed literature, followed by our conclusion.

## 2. Methods

We searched the following databases from 1965 to 2014 (January 1, 2014) for articles published in English in PubMed and Google Scholar. We used a compound search strategy combining abbreviated and non-abbreviated equivalent terms to identify R&D, such as ‘‘R&D”, “Research and Development” included in various expressions. We followed the ‘‘related citations’’ and ‘‘cited by’’ links in Google Scholar and PubMed to identify potentially relevant studies. In addition, we carefully went through all bibliographies of all included articles and closely related but excluded studies to search for additional relevant citations. We used the following search terms: “Determinants of pharmaceutical research and development”, “Pharmaceutical research and development productivity”, “Pharmaceutical research and development commercialization”. The search was restricted to diagnostic laboratory facilities for studies on humans only. Studies were included, if they clearly fell within the three areas we consider relevant for an R&D implementation (productivity, commercialization and expenditures). No merit score has been added.

## 3. Results

**Figure 1:**
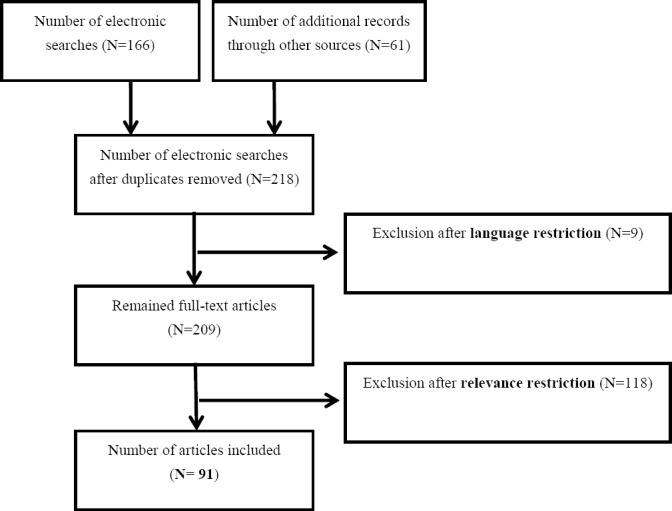
Procedure to include or exclude articles in the systematic review

The search generated a total of 218 articles and reports that were examined. However, 91 sources were considered appropriate and used for further analysis. Figure 1 provides details of our inclusion and exclusion criteria following the initial search performed on January 01, 2014. The figure is adapted from the flow diagram proposed by the Preferred Reporting Items for Systematic reviews and Meta-Analyses (PRISMA) (Moher et al., 2009).

In quest of strategies needed to revamp R&D outputs, we identified different literature sources relevant to productivity, R&D commercialization, and cost-effectiveness of R&D expenditure. Sources for each category were 12, 23 and 11, respectively. The setting of these three areas was optimal in organizing and evaluating the determinants of R&D expenditures.

### 3.1 Productivity of an R&D system

Pharmaceutical industry may harbor an R&D system that may disclose productivity and be of extreme value, but it depends from the investments (Paul et al., 2010). An R&D strategy may not be worthwhile, unless efficiency and effectiveness are intensified. Specifically, efficiency refers to the ability to achieve the maximum output (e.g. new molecular entity releases) with inputs (e.g. R&D expenses), while effectiveness tells how the output leads to greater outcomes, i.e. how valuable the drugs are in terms of improving the quality of life of the patients (Paul et al., 2010). Effectiveness could be achieved through cost-effectiveness of new medicines, which must consider the perceived value of the medicine. Measurement of cost-effectiveness is centred on the valuation of an individual’s preference and can be obtained via cost-utility analysis approaches (Ament et al., 2014).

From time to time, pharmaceutical industry may face an increase of productivity crisis as attested by a wide range of sources (Pammolli et al., 2011; DiMasi, 2014; Higgins & Rodriguez, 2006). Pharmaceutical companies are affected irrespective of their geographic location. The productivity crisis affects both European and North American (United States and Canada) companies alike (Pammolli et al., 2011). In some reports, it has been suggested that some decline in productivity might be due to a rising concentration in R&D activities, particularly in areas showing a theoretical high risk of failure. Under the crisis, as internal productivity ceaselessly declines and nervousness seems to set in, companies may start to look for a way out in an effort to restock their research pipelines.

The challenge of combating the crisis by keeping productivity high is a priority to pharmaceutical companies. They are progressively looking for collaboration opportunities with other companies (e.g., venture companies) and other organizations (e.g., universities), so that more new drugs arising from the knowledge of basic sciences and human pathology as well as implementing potential clinical trials can be produced (Higgins and Rodriguez, 2006; Dimasi et al, 2014; Tsukamoto & Kaneko, 2013). Actually, collaboration involving proper use of external resources, such as highly qualified personnel from other relevant institutions, can bring additional value to the company in terms of improved activities and increased revenue (Danzon et al., 2005; Veugelers, 1997). Most especially, some authors found that products developed in collaboration with other institutions tend to have a higher probability of success among larger firms, at least for phase II and phase III clinical trials. A study from the United States examined the impact of pharmaceutical and biotechnology companies entering into collaborative and shared innovation approaches (e.g., joint development, mergers and acquisition strategies, and in-licensing) by looking into the development histories of new drug entities that obtained approval (Dimasi et al, 2014).

Boosting R&D Productivity entails commitment to increase the number of innovative new medicines, which need also to be cost-effective. A set of strategies ought to be implemented to trim down costs including a model parameters cost reduction and sensitivity analyses, which can reduce costs per new molecular entity (NME) by 28% and 50%, respectively (Hagen-Zanker et al., 2012; Moher, 2009; Paul et al., 2010). The potential beneficial effect on capitalized cost per NME launch results from improvements in a number of R&D parameters, including cost, cycle time (CT) and probability of technical success. It has been suggested that a 28% reduction in cost per NME could be achieved through the aggregate actions resulting in a 9% reduction in CT from lead optimization to Phase I. Moreover, a 10%, 15% and 20% reduction in cost could be achieved for clinical development Phases I, II and III, respectively. An even larger 50% reduction in cost per NME could be considered by additionally reducing CT by 20% across Phases II and III, a further remarkable reduction in cost by 10% across all phases, and an increase in Phase III by 14% without negatively affecting other parameters (Paul et al., 2010).

An efficient regulatory relationships process and a refined tax credit system are also critical as source of incentives to strengthen productivity as identified in some reports. Interestingly, this seems to be true irrespective of company size (DiMasi et al., 2014). There was a high increase in the issuing of the number of non-compliance notices over 1997-2000 that affected some companies in marketing determined products (Li & Tomalin, 2002). Improvement of the tax credit policy can be a motivation to companies involved in R&D. A Canada-USA comparison of R&D tax credits revealed that, in Canada, expenses in R&D that are eligible for tax credit are actually very limited (Markman et al., 2008).

### 3.2 Pharmaceutical Ideas Commercialization

R&D facilities want indeed to earn returns from their discoveries. The need for the proper commercialization of research even grows higher, in the event of budgetary constraints. Of the 23 articles reviewed under this section, 11 referred to the current modes of commercialization and 12 to the ensuing strategies that should be followed to guide R&D implementation. It may also be useful to set up “think-tank” events that may help engaging about novel applications of existing science and technology, identifying issues and gaps in current knowledge, and proposing solutions.

#### 3.2.1 Types of Commercialization

A number of sources have acknowledged the many ways through which university research could be exploited (Lowe, 1993; Markman et al., 2008). Actually, different types of commercialization require different mobilization strategies during implementation. Hence, a taxonomy has been developed, which comprises three modes of commercialization: internal, quasi-internal (e.g., incubators), and externalization approaches (Markman et al., 2008).

With regard to internal approaches, universities set in place measures to handle tensions that may occur between faculty and the commercial clients by establishing specialized structures. An example of these specialized institutions is so-called Technology Transfer Offices (TTOs) run by university technology managers. Regarding quasi-internal approaches, universities use structures such as business incubators that work to facilitate the coming together of important players skillful at speeding up technology commercialization (Phan et al., 2005). Externalization approaches include university research parks, regional clusters, academic spin-offs and start-ups, licensing, contract research and consultancy, joint venture spin-offs and open science, and innovation. University research parks host together in one-location organizations of various entities with the aim of favoring positive networking. Regional clusters are means often put in place by some universities away of the metropolitan area to overcome resource endowment difficulties and enhance their economic scale (Markman et al., 2008). Academic spin-offs and start-ups are new ventures formed for the licensing or assignment of intellectual property rights. Licensing is a way for firms to improve the impact of their discoveries by selling licenses to other firms (Thursby and Thursby, 2007). Through contracts, firms may generate research and consultancy revenues. Joint venture spin-offs are alliances and collaborations often involving a company or a university that jointly own a technology. Open innovation gives the opportunity to various stakeholders (e.g., customers, communities, agents) to meet (e.g., online) and together innovate and create. In fact, the so-called “think-tank” events may be crucial in engaging about novel applications of existing science and technology, identifying issues and gaps in current knowledge, and proposing solutions.

Internal approaches via TTOs often place greater emphasis on revenue maximization. However, examination of the extent of commercialization of university research by means of TTOs yielded meager results. In fact, an empirical study in the USA found that 40 percent of responding TTOs earned lower than $600,000 without yet accounting for the salaries of TTO personnel (Thursby and Thursby, 2008; Thursby and Thursby 2005). Legal fees need also to be properly included. Practically, the problems facing TTOs include high transaction costs to carry new scientific discoveries to the marketplace (Litan et al., 2008). TTOs are much regarded as monopolies without having always incentives in maximizing the actual number of commercialized discoveries. Technologies with the largest and quickest returns could be favoured to the detriment of technologies with much longer-term benefits to society.

Therefore, an initiative was started, which stood against models that placed greater credit to the maximization of licensing income (Litan et al., 2008). Proponents of this initiative suggested an interesting “volume model”, which singularly stresses the number of university innovations and rapidity of the product in the marketplace. Volume models share some common features, including provision of incentives for bringing innovations into the marketplace besides returns collection, recognition of faculty as the key agents of innovation and commercialization, and emphasizing standardization in the interactions of campuses with their faculty and with industry.

There are four brands of volume models identified in our literature review, including free agency, regional alliances, internet-based approaches, and faculty loyalty (Thursby & Thursby, 2005). *Free agency* goes with an agreement that faculties return some portion of their profits to the university, as they are authorized to choose a third party to negotiate license arrangements for entrepreneurial activities. Under *regional alliances*, multiple universities take advantage of the economy of scale to diminish transaction costs at commercialization by constituting consortia that develop their own mechanisms for commercialization. *Internet-based approaches* maximize volume by creating a setting whereby entities work to generate new ideas. A number of implementers may come together to refine the ideas. Regarding *faculty loyalty*, universities forego their intellectual property rights, while implicitly expecting some donation from the loyal faculty member consider up the fruits of their success back to the university.

#### 3.2.2 R&D Strategies Relevant for Commercializing New Ideas

Irrespective of the type of commercialization, it has been suggested that emphasis has to be put on strategies that emerge from the literature as to how laboratories should commercialize new technologies. Of the 12 articles reviewed, these strategies often include reliability of companies on “sourcing-in” to boost own R&D strategies (3 articles), enhancement of researchers’ involvement in commercializing own findings (2 articles), strengthening the importance of social and network factors (3 articles), and emphasis on open innovation approaches (4 articles).

Regarding the reliability of companies on “sourcing-in” to boost own R&D strategies, there is a growing tendency of universities to do patenting and licensing of discoveries generated by their research staff. They are buying or licensing discoveries from other companies (Grabowski, 2011). Licensing is indeed beneficial, because it generates more profits, improves time to market, trims down R&D costs, and, ultimately, offers ways to penetrate new markets (Markman et al., 2008; Markman et al., 2009). At the same time, companies are outsourcing some unused internal inventions to smaller firms or research laboratories often within universities. Thus, a new market setting seems to have evolved offering companies a window of opportunity to fund their R&D activities. Companies are increasingly becoming more market-inclined entities by participating in the improved “market for research ideas”.

The degree of faculty-industry engagement is critical, especially if attention relies on the enhancement of researchers’ involvement in commercializing own findings. This can be formal through TTOs or informal using different channels (Siegel et al., 2004). Often a non-negligible portion of patents with public university faculty inventors goes to firms and not to the corresponding public university (Thursby & Thursby, 2005). More involvement of the faculty is likely to ensure proper follow-up during the process of commercialization.

Strengthening the importance of social media and networks and factors of success is known as relevant strategy for commercializing new ideas. The professional relationships of university researchers with their peers from within campus and outside the academic milieu give them a comparative advantage to successfully connect with the industry for the economic exploitation of their scientific discovery (Litan & Reedy, 2008). Typically, universities seek to increase ownership of research and commercialization. Some studies noted an increase in university patenting (Coupe, 2003), while a number of studies underline the successful increase in proliferation of university spin-offs and research parks (Mowery et al., 2001).

Last, but not the least of suggested commercialization strategies is the emphasis on open innovation approaches. The world is experiencing an expansion of online social networks following the advent of ICTs. This progress has prompted research firms to increasingly become more open to innovations by increasingly involving external actors in the conduct of their own R&D activities (Chesbrough, 2003; Enkel et al., 2009). From external actors, firms can find the extra expertise they need to raise the chances of bringing new ideas to the market place. Some innovation-seeking agencies take advantage of the available online means of communication to refine their ideas online with other network members who can add their own contributions to become co-developers (Markman et al., 2008). CAST-Center for Academic Spin-offs Tyrol, Gründungszentrum GmbH is an example active in Austria (http://www.cast-tyrol.com). An improved gain in time and sophistication in the refinement of the research idea can be achieved in this way. Not all firms open up to innovation to the same extent, however. A study conducted in Italy found different degrees in openness to innovation according in the types of external partners to the firm and the steps in the innovation process (Lazzarotti et al., 2010).

### 3.3 Determinants of R&D Expenditures

In order to have proper control over the investments in R&D, in-depth understanding of the factors influencing expenditures is critical. Table 1 shows an inventory of the significant determinants of R&D expenditures from our literature review. Each of the nine studies included was based on a regression model and covered a period within the timeline indicated above. We describe the determinants in terms of their correlation sign with the R&D expenditure variable, the study period, the chosen dependents, the author(s) and year of publication, and the location of the study. The correlation sign is that of the coefficient (research elasticity) on the explanatory factor, i.e. the determinant, of the dependent variable R&D expenditure. We focused only on the direction of the associations without including their magnitudes. Only the relevant articles from our pool of searched articles have been included. A relevant article was the one that examined factors influencing an R&D expenditure variable or its proxies in the framework of an empirical study. Common R&D expenditure proxies from the literature included: growth in firm-level absolute R&D expenditure variable (Mitchell et al., 1995), R&D expenditures (Malmberg, 2008), change in R&D intensity (R&D divided by Sales) (Mitchell et al., 1995; Giaccotto et al., 2005; Siddharthan, 1992; Grabowski & Vernon, 2000; Mahlich & Roediger-Schluga, 2006), other ratios such as R&D divided by Capital Assets (Trushin, 2011), and the number of new drugs in development to target an illness.

**Table 1:** Inventory of Significant Determinants of R&D from Regression Model Studies in Pharmaceutical Firms (N=9)

Determinants of R&D	Correlation Sign	Study Period	R&D Proxy	Author(s) and year of publication	Location
Firm size^1^	Negative	1975-1990	Growth^11^	Mitchell et al. (1995)	Japan
Firm size (by sales)	Positive	1992-1993	IRD^10^	Veugelers (1996)	Finland

Profitability Rate^2^	Positive	1975-1990	Growth	Mitchell et al. (1995)	Japan
Firm’s lag profitability	Positive	2003-2010	R&D expenses	Simanjuntak and Tjandrawinata (2011)	USA
Industry margin^3a^	Positive	1987-1998	R&D/Sales	Mahlich and Roediger-Schluga (2006)	Japan
Industry margin	Positive	1974-1994	R&D/Sales	Grabowski and Vernon (2000)	USA
Foreign sales rate	Positive	1980-2001	Change in R&D intensity	Giaccotto et al., 2005	USA
Sales from new drugs	Positive	1987-1998	R&D/Sales^12^	Mahlich and Roediger-Schluga (2006)	Japan
Sales from new drugs	Positive	1974-1994	R&D/Sales	Grabowski and Vernon (2000)	USA
Sales to assets	Negative	1997-2007	R&D/Assets	Trushin (2011)	11 OECD
Sales expected	Positive	1960-1990	Current year R&D expenses	Malmberg (2008)	Sweden
Drug sales rate	Negative	1975-1990	Growth	Mitchell et al. (1995)	Japan

Cash flow	Positive	2003-2010	R&D expenses	Simanjuntak and Tjandrawinata (2011)	USA
Cash flow from past (i.e. Internal funds)	Positive	1960-1990	Current year R&D expenses	Malmberg (2008)	Sweden
Cash flow to sale	Positive	1974-1994	R&D/Sales	Grabowski and Vernon (2000)	USA
Cash flow to sale	Positive	1987-1998	R&D/Sales	Mahlich and Roediger-Schluga (2006)	Japan
Cash flow to assets	Positive	1997-2007	R&D/Assets	Trushin (2011)	11 OECD

Cash holding to assets	Negative	1997-2007	R&D/Assets	Trushin (2011)	11 OECD
R&D to assets at *t-1*	Positive	1997-2007	R&D/Assets^13^	Trushin (2011)	11 OECD^11^
R&D expenses at *t-1*	Positive	1992-1993	IRD	Veugelers (1996)	Finland
Foreign Equity^3b^	Positive	1975-1990	Growth	Mitchell et al. (1995)	Japan
Start-up date	Positive	1975-1990	Growth	Mitchell et al. (1995)	Japan
Real drug price	Positive	1980-2001	Change in R&D intensity	Giaccotto et al., 2005	USA
Population^4^	Positive	1998-2000	R&D expenses	Li (2002)	Canada
Firm’s Chemical division	Positive	1992-1993	IRD	Veugelers (1996)	Finland
Firm’s IT division	Positive	1992-1993	IRD	Veugelers (1996)	Finland
Public subsidies at *t-1*	Positive	1992-1993	IRD	Veugelers (1996)	Finland
Collaboration^5^	Positive	1992-1993	IRD	Veugelers (1996)	Finland
Acquisition expenses^6^	Positive	1992-1993	IRD	Veugelers (1996)	Finland
R&D intensity	Positive	2003-2010	R&D expenses	Simanjuntak and Tjandrawinata (2011)	USA
Price-to-book ratio	Negative	1997-2007	R&D/Assets	Trushin (2011)	11 OECD

1 In terms of R&D expenditures;

2 Net Profit divided by Net Revenue;

3a Weighted average profit rate of the entire industry calculated as pre-tax profits divided by sales;

3b Rate of shares owned by foreign investors;

4 Provincial population for all provinces and territories in Canada;

5 Firm’s engagement in R&D cooperation (Company with absorptive capacity);

6 Expenditures for technology acquisition embodied in equipment and for licensing external technology in previous year (Company with absorptive capacity only);

7 Growth in firm-level absolute R&D expenditure;

8 R&D expenditure divided by sales;

9 Ration of R&D to total firm assets;

10 IRD is internally financed intramural expenditures for R&D;

11 There were 11 OECD countries involved in the study.

Regardless of the R&D expenditure proxies, the recurrent determinants of R&D expenditures from previous studies included cash flow, sales, profitability rate, and firm size. Not all determinants always shared the same correlation patterns with the dependent variable – R&D expenditure – across studies. The “cash flow” variable occurred in five studies from three different continents (America, Europe, and Asia) and exhibited a positive correlation with the variety of proxies used in studies. The *profitability ratio* showed a similar correlation pattern (positive sign) with the different proxies across studies from Japan and US. Conversely, for the *sales* variable, which occurred in six studies from across the world, one of the studies involving 11 OECD countries exhibited a negative correlation with the R&D expenditure proxies. Another determinant with inconsistent correlations is *firm size*, which was negative for the study from Japan where the proxy was R&D expenditure growth and positive for the study from Finland where the proxy was the amount of internal funds spent on R&D.

## 4. Discussion

To be able to take valuable new ideas to their targeted market as effectively as possible is of primary importance. Pharmaceutical companies aim to improve upon earnings and most preferably their profit margins. It was evident that adequate and wise cost restriction may secure substantial profit margins. This is an incentive to manage pharmaceutical firms for accrued investment in R&D, thereby increasing productivity in a number of profitable projects. According to our analysis, it seems that implementation of an R&D strategy that is built on effective cost handling and refined commercialization for profit improvement will open avenues for resources to mitigate escalating health care costs.

With the evolution of refined business models, pharmaceutical firms can make R&D cost-effective by increasing cooperation with university laboratories. They can further through cooperation with peer firms reduce the economic risk involved in delving into R&D initiatives; thereby making R&D investments more attractive. Classical business models whereby contacts between firms and universities were often undertaken through subsidiaries are already becoming gradually outdated (Deventer, 2005). Pharmaceutical firms nowadays approach clinical researchers early in the drug development process, so as to keep with time schedule and to have access to patient and special facilities. With increasing budgetary constraints, clinical researchers with promising ideas henceforth enter into agreement with pharmaceutical companies for further development. Indeed, an innovative business model in terms of a frequent bilateral collaboration between the industry and academic centers during drug discovery, development and regulatory phases is worthwhile.

In order to meet cost-effectiveness in R&D expenses, caution is, however, required for two reasons. First, there are manifold determinants of R&D expenditure in the pharmaceutical industry literature to account for with often-mixed outcomes of some determinants of R&D in empirical studies. Second, some determinants yielded inconsistent correlation outcomes across studies (e.g., sales and firm size), whereas cash flow and sales were consistently positive across different countries. Inconsistency in outcomes may arise owing to different reasons: cross-sectional differences across industries, conflicting outputs in the pharmaceutical industry, and research environment differences across different years of study (Mitchell et al., 1995). Specific characteristics of regression analyses inherent to each of the examined studies might have played a role. Actually, a review by Hall, Mairesse and Mohnen (2010) comprising both cross-sectional and panel studies found high variation in R&D elasticity of output across different types of estimation: from 0.01 to 0.25. The cross-sectional estimates were indeed higher than the temporal estimates. Consistency in outcomes of both cash flow and profitability rate is also useful to underline one fact: implementation of R&D strategy that builds on effective cash handling to rein in costs also benefits from sustained profit.

Moreover, it is worth pointing out that, based upon own strategic plans some pharmaceutical companies may purchase smaller firms in order to improve their economy of scale and scope. By acquiring smaller firms, these pharmaceutical companies want to achieve an increase in the number of profitable R&D projects or face the need to reduce R&D costs. Although this approach aims at cutting R&D expenditure to boost profit, it entails underlying extra transaction costs in the short run at least. For instance, the cost of transacting information across the structures of a firm in different regions should never be ignored, especially during the new drug application process (Hamada S, Shibata A, Urushihara et al., 2013). Strategic acquisition of smaller firms often requires that pharmaceutical company may need to invest resources and time to research on the targeted small firm. This proceeds from acquisitions that are positively correlated with prior access to information about the research and development activities at the smaller firms (Danzon, 2006; Higgins and Rodriguez, 2006). Thus, there may be some transaction cost involved; because of overbidding and wrong selection. Poor integration can indeed be detrimental to the acquirer. Components of R&D expenditure in one situation may not apply in another. However, improved profit will motivate additional investments in research and development.

### 4.1 Towards Implementation of a Profitable R&D System

**Figure 2:**
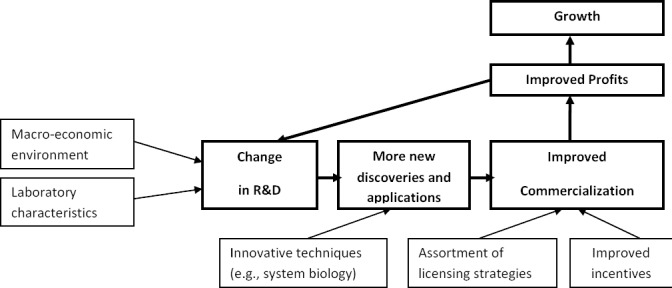
Framework for the implementation of an R&D investment strategy

A successful R&D implementation strategy should enable the growth of firms or laboratories of all sizes (Figure 2). Better handling of the investments in R&D will improve profits through enhanced productivity supplemented with refined marketing strategies and sales (Scherer, 2001; Siddharthan, 1992; Ito & Pucik, 1993). Better handling occurs when R&D investments are cost-effective and attempt to minimize costs of drug discovery, while valuing the perceptions of users regarding the drug.

Cost-effectiveness and ultimate value of the intervention both shape the cost-utility notion (Ament et al., 2014; Richardson, 1994; Redelmeier & Weinstein, 1999). To evaluate accuracy, proper understanding of the determinants of R&D investments is mandatory. Laboratory characteristics such as focused rather than broad experience and alliances with other firms are some examples (Amir-Aslani A. and S. Negassi, 2006; Henderson R and Cockburn; 1996). Investment in R&D is likely to boost the quantity of new discoveries and applications that must be marketed and sold. Improving scientific and management decisions can make launching of successful drug candidates more economically attractive in terms of time and money, which will uphold productivity (Bains, 2004). Fostering new technical approaches such as system biology (i.e., orderly combination of technologies for better data and information) is likely to ameliorate the drug discovery process (Naylor, 2004; Davidov et al., 2003). As well, expansion and greater adoption of modeling & simulation techniques (Hunt et al., 2013), and drug re-purposing methods (Dudley et al., 2011), are additional approaches to boost the quantity of new discoveries.

Improved commercialization yields more revenues and more profits. Both accrued actual and expected profits encourage firms to invest more on R&D to achieve lasting growth (Simanjuntak & Tjandrawinata, 2011; Chander & Aggarwal, 2007). Commercialization outcomes (e.g. revenue, start-up creation and marketing route) are dependent upon the diversity of licensing strategies, autonomy of TTOs, sharing of revenues with scientists and adequate compensation of TTOs officers (Lowe, 1993; Markman et al., 2008).

Finally, more investment in R&D proceeds through better productivity and improved commercialization to ultimately enhance growth *ceteris paribus* (Cameron, 1998). There is mixed empirical evidence whether R&D outlays always lead to firm growth, especially when firms are considered in aggregate. Only when factors such as firm size, patenting, and persistence in patenting are accounted for, is a positive influence of R&D on growth obvious among small firms (i.e., less than 500 employees) compared to large firms (Demirel & Mazzucato, 2012).

### 4.2 Conclusion

In conclusion, handling R&D determinants may require caution, but we would like to emphasize that implementation of R&D systems is indeed in line with productivity, if it reflects dual embodiment of efficiency and effectiveness. More investment in R&D proceeds through better productivity and improved commercialization enhancing the growth. Productivity crisis can be mitigated via more efficient regulatory process, improved tax credit policy, and collaboration not just for increased new technologies, but also for regional and global policies in favor of fairer drug business models. To supplement productivity, adapted and profitable commercialization strategies, a wide range of marketing, and sales options are available. A properly timed commercialization process that is supported by both favorable patenting regulation and tax credits measures should be integral part of an R&D investment plan. Encouraging collaboration across R&D facilities of various scales and entities is likely to add value. This can be done through increased extra returns from placement of research output and strengthened partnership between universities and pharmaceutical industry. Doing so will create a guaranteed liquid market for research ideas beneficial to scientists of both big and small R&D facilities.

R&D determinants often exhibited an inconsistency in outcomes when considering the patterns of association with the dependent variable – R&D expenditures. Variations in correlation signs of the same R&D expenditure determinant often occurred across studies. Hence, to ensure cost-effectiveness of R&D initiatives and thereby improved profitability of research and development entities, the determinants of R&D expenditures from previous studies may be wisely integrated in the implementation project plan. Accounting for each specific implementation context, therefore, becomes critical. By providing evidence, the systematic review will assist research laboratories in the design of cost-saving technologies that can ultimately add more value to the healthcare system. Health care plan makers may pinpoint an implementation framework based on this systematic review. This framework may be able to emphasize the opportunity to set up more cost-effective R&D systems in the future of healthcare institutions.
